# Which T Category of Nasopharyngeal Carcinoma May Benefit Most from Volumetric Modulated Arc Therapy Compared with Step and Shoot Intensity Modulated Radiation Therapy

**DOI:** 10.1371/journal.pone.0075304

**Published:** 2013-09-25

**Authors:** Ying Sun, Rui Guo, Wen-Jing Yin, Ling-Long Tang, Xiao-Li Yu, Mo Chen, Zhen-Yu Qi, Meng-Zhong Liu, Jun Ma

**Affiliations:** State Key Laboratory of Oncology in Southern China, Department of Radiation Oncology, Sun Yat-sen University Cancer Centre, Guangzhou, China; The Chinese University of Hong Kong, Hong Kong

## Abstract

**Background:**

To compare volumetric modulated arc therapy (VMAT) with conventional step and shoot intensity modulated radiation therapy (s-IMRT) in nasopharyngeal carcinoma (NPC) patients, and identify which T category patient gains the maximum benefit from VMAT.

**Methods:**

Fifty-two patients that randomly selected from 205 patients received VMAT at a single center were retrospectively replanned with s-IMRT. For a fair comparison, the planning target volume (PTV) coverage of the 2 plans was normalized to the same level. A standard planning constraint set was used; the constraints for the organs at risk (OARs) were individually adapted. The calculated doses to the PTV and OARs were compared for s-IMRT and VMAT plans generated using the Monaco treatment planning system.

**Results:**

VMAT and s-IMRT plans had similar PTV coverage and OAR sparing within all T categories. However, in stratified analysis, VMAT plans lead to better or similar sparing of the OARs in early T category patients; and lead to poorer sparing of the OARs in advanced T category patients (*P*<0.05). VMAT shows significant advantages for low dose burden (P<0.05) compared with s-IMRT. The delivery time per fraction for VMAT (424±64 s) was shorter than s-IMRT (778 ± 126 s, *p*<0.01).

**Conclusions:**

VMAT provides similar dose coverage of the PTVs and similar/better normal tissue sparing in early T category NPC, and poorer OARs sparing in advanced T category NPC. And VMAT shows significant advantages for low dose burden and delivery time.

## Introduction

Nasopharyngeal cancer (NPC) has an extremely unbalanced endemic distribution, with the area of highest incidence centered in southern China [1]. Radiation therapy is the mainstay of treatment modality for non-metastatic NPC, due to its anatomic features and biological behavior. The introduction of intensity-modulated radiation therapy (IMRT) exited radiation oncologists, as it can deliver a high dose to the tumour target while significantly reducing the dose to the surrounding normal tissues [2]. Retrospective and prospective studies have confirmed that IMRT improves disease control and reduces the treatment toxicity profile in NPC [3,4].

Despite its advantages, a number of issues related to IMRT techniques remain unaddressed [5]. Firstly, the prolonged beam delivery time may reduce treatment accuracy due to increased intra-fractional patient motion and biological effects [6]. Additionally, the high number of monitor units (MUs) required increases the peripheral dose, which may potentially increase the incidence of radiation-induced secondary cancer [7,8].

To overcome the drawbacks of IMRT, so-called volumetric-modulated arc therapy (VMAT), proposed by Yu in 1995, has received renewed interest due to the introduction of linear accelerator delivery control systems [9]. VMAT enables variation in the ML leaf position, dose rate and gantry rotation speed with continuous modulation during a single 360° rotation [10]. VMAT may offer dosimetric advantages in head and neck cancer [11-13]. However, there are few reports of VMAT treatment planning in NPC, which is an ideal advanced benchmark for the assessment of conformal avoidance during radiotherapy, as the anatomic features of NPC require highly sophisticated techniques to ensure adequate treatment. Furthermore, treatment planning for the different T categories of NPC is extremely difficult, and it remains unknown whether VMAT is comparable to fixed field IMRT for the treatment of the different T categories of NPC.

This study aimed to compare the dosimetric parameters of VMAT and step and shoot IMRT (s-IMRT) treatment plans in NPC patients, in order to comprehensively evaluate the benefits of the VMAT technique in different T categories of NPC.

## Methods

### Patient selection and staging evaluation

Between August 2010 and June 2011, 52 biopsy-proven, non-metastatic NPC patients were randomly selected from 205 patients received VMAT at a single center (male/female ratio 4.2:1, 42 men/10 women; median age 46 years, range 21–71 years). Histological examination confirmed all patients had World Health Organization (WHO) type II or III disease. Approval for retrospective analysis of the patient data was obtained from the ethics committee of Sun Yat-sen University Cancer Center. All patients have signed written consent for the radiation treatment and were informed the technique for radiation, the possible side effects, the risk and so on.

The pre-treatment evaluations included a complete physical examination, hematologic and biochemistry profiles, fibrotic endoscope examination of the nasopharynx, and MRI or contrast-enhanced computed tomography of the nasopharyngeal and cervical region to evaluate the primary tumour extent and regional lymph node involvement. Chest radiography, bone scintigraphy and abdominal region ultrasonography were used to exclude distant metastases.

All patients were staged according to the American Joint Committee on Cancer (*Manual for Staging of Cancer, 7th* edition) system. The T-classification distribution was T1, 19.2%; T2, 19.2%; T3, 40.4% and T4, 21.2%. The overall stage distribution was stage I, 9.6%; stage II, 25.0%, stage III 42.3% and stages IVA and IVB, 23.1%.

### Simulation and immobilization

All patients were immobilized in the supine position using a head neck and shoulder thermoplastic mask. CT was performed after administration of intravenous contrast medium; 3 mm slices were obtained from the head to 2 cm below the sternoclavicular joint.

### Target volume delineation and dose prescription

The target volumes were defined in accordance with the International Commission on Radiation Units and Measurements reports (ICRU) 50 and 62. The gross tumor volume (GTV) was determined from the MRI, clinical information and endoscopic findings. The clinical target volumes (CTV) were individually delineated on the basis of tumor invasion [14]. The first clinical tumor volume (CTV-1) was defined as the GTVnx with a 5–10 mm margin, to include high-risk regions of microscopic extension encompassing the whole nasopharynx. The second CTV (CTV-2) was defined as CTV-1 with a margin of 5–10 mm, for low-risk regions of microscopic extension; this margin could be reduced if it was in close proximity to critical structures. The organs at risk (OAR) included the brainstem, spinal cord, temporal lobe, optic nerves, optic chiasm, lens, eyes, parotid glands, mandible, temporo-mandibular joint, middle ear and larynx.

The prescribed dose was 70 Grey (Gy) to the PTV of GTVnx, 60 Gy to the PTV of CTV-1, 56 Gy to the PTV of CTV-2 and 60–68 Gy to the PTV of GTV for involved cervical lymph nodes, in 33 fractions. Treatment was delivered once daily, over 5 fractions per week. All targets were treated simultaneously using the simultaneous integrated boost (SIB) technique.

### Planning objectives and techniques

A standard constraint set, with reference to RTOG0615, was used for optimization and evaluation. The PTVs of each target were created by adding a 3 mm 3D margin to the delineated target volume to compensate for treatment set-up variability and internal organ motion. The planning constraints and objectives for the PTVs are presented in Table 1. The aim was to achieve coverage of 95% of any PTV with ≥ 100% of the prescribed dose. No more than 20% of any PTV was to receive > 77 Gy (equivalent to 110% of the PTVnx dose). The planning OARs volumes (PRVs) were defined (Table 2) by adding a 3 mm safety margin to all structures, except for the spinal cord (5 mm margin).

**Table 1:** Dosimetric comparison of VMAT and IMRT for the PTVs in 52 NPC patients.

			**Pass-rate**			**Mean Dose (SD**)			
**Target**	**Objective**		**IMRT**	**VMAT**	***P* value**	**Index**	**IMRT (Gy**)	**VMAT (Gy**)	***P***
**PTV_7000**	V93%^¶^	≥99%	98.1%	98.1%	1	D2^§^	75.96 (1.03)	77.14 (1.39)	0.13
	V100%^*^	≥95%	96.2%	96.2%	1	D50^&^	73.90 (0.68)	73.63 (0.75)	0.013
	V110%^†^	≤20%	100%	100%	1	D95^‡^	70.96 (0.70)	70. 61 (0.55)	<0.001
	V115%^║^	≤5%	100%	100%	1	D98^#^	69.85 (1.39)	69.41 (1.02)	<0.001
						CI	0.54 (0.13)	0.48 (0.15)	<0.001
						HI	1.07 (0.02)	1.09 (0.02)	<0.001
**PTV_6000**	V93%^¶^	≥99%	100%	100%	1	D2^§^	75.62 (0.95)	76.83 (1.36)	<0.001
	V100%^*^	≥95%	100%	100%	1	D50^&^	71.47 (1.18)	71.35 (1.53)	0.001
						D95^‡^	64.69 (1.72)	64.50 (1.24)	<0.001
						D98^#^	62.97 (1.35)	62.85 (1.36)	0.41
						HI	1.16 (0.04)	1.18 (0.03)	<0.001
**PTV_5600**	V93%^¶^	≥99%	96.2%	91.7%	0.431	D2^§^	74.47 (1.19)	75.31 (1.38)	<0.001
	V100%^*^	≥95%	96.2%	92.3%	0.763	D50^&^	63.51 (1.63)	62.39 (1.64)	<0.001
						D95^‡^	57.31 (0.92)	57.03 (0.81)	0.01
						D98^#^	55.61 (1.31)	54.70 (1.25)	<0.001
						HI	1.28 (0.05)	1.30 (0.03)	0.07

Abbreviations: PTV, planning target volume; HI, homogeneity index; CI, conformity index; IMRT, intensity modulated radiation therapy; VMAT, volumetric modulated arc therapy.

¶ Percentage dose covering 93% of the PTV

* Percentage dose covering 100% of the PTV

†: Percentage volume that received > 110% of the Rx (prescribed dose)

║ Percentage volume that received > 115% of the Rx (prescribed dose)

§ Dose received by 2% of the volume

&: Dose received by 50% of the volume

‡: Dose received by 95% of the volume

#: Dose received by 98% of the volume

**Table 2:** Dosimetric comparison of VMAT and IMRT for the organs at risk (OARs) in 52 NPC patients.

			**Pass Rate**				**Mean dose (SD**)		
**OAR**	**Objective**		**IMRT**	**VMAT**	***P***	**Index**	**IMRT(Gy**)	**VMAT(Gy**)	***P***
SpinalCord	Max	≤45Gy	82.7%	80.8%	1	max	42.09 (6.53)	45.12 (4.19)	<0.001
SpinalCord_PRV	D1^¶^	≤50 Gy	100%	100%	1	D1^¶^	43.33 (3.79)	45.40 (3.65)	<0.001
BrainStem	Max	≤54Gy	28.8%	32.7%	0.77	max	58.13 (5.92)	58.19 (6.45)	0.83
BrainStem_PRV	D1^¶^	≤60Gy	69.2%	65.3%	0.77	D1^¶^	58.22 (5.78)	57.99 (6.43)	<0.001
OpticNerves_L	Max	≤50Gy	61.5%	57.7%	0.73	Max	35.32 (17.92)	36.38 (18.68)	0.32
OpticNerves_L PRV	D1^¶^	≤54Gy	69.2%	65.4%	0.73	D1^¶^	43.25 (18.02)	45.79 (18.35)	0.029
OpticNerves_R	Max	≤50Gy	59.6%	65.4%	0.37	Max	35.66 (17.19)	36.84 (17.44)	0.076
OpticNerves_R PRV	D1^¶^	≤54Gy	69.2%	65.4%	0.73	D1^¶^	43.25 (18.02)	45.79 (18.35)	0.017
Chiasm	Max	≤50Gy	50.0%	53.8%	0.69	Max	43.77 (14.61)	45.09 (15.36)	0.22
	D1^¶^	≤54Gy	59.3%	55.6%	0.76	D1^¶^	44.03 (17.16)	45.82(18)	0.01
Lens_L	Max	<25Gy	100%	100%	1	Max	7.59 (3.11)	8.25 (3.36)	<0.001
Lens_R	Max	<25Gy	100%	100%	1	Max	7.80 (3.26)	8.38 (3.32)	0.008
Parotid _L	Mean	< 26 Gy	0	0	1	Mean	37.68 (5.05)	38.15 (5.40)	0.22
	V30^§^	<50%	28.3%	28.3%	1	V30^§^	62.17 (16.63)	63.67 (17.94)	0.22
	V20^*^	>20cc	5.8%	7.7%	1	V20^*^	3.35 (3.59)	3.46 (4.16)	0.84
Parotid _R	Mean	< 26 Gy	0	0	1	Mean	38.44 (6.08)	39.39 (6.61)	0.032
	V30^&^	<50%	28.3%	28.3%	1	V30^§^	64.31 (18.60)	65.63 (18.71)	0.30
	V20^*^	>20cc	5.8%	7.7%	1	V20^*^	3.40 (4.55)	3.70 (5.67)	0.61
TemporalLobe_L	Max	≤60Gy	7.7%	9.6%	1	D1^¶^	60.41 (7.22)	60.25 (7.84)	0.82
TemporalLobe_R	Max	≤60Gy	3.8%	3.8%	1	D1^¶^	60.44 (10.58)	62.31 (8.05)	0.11
Mandible_L	D1cc^&^	<75Gy	100%	100%	1	D1cc^&^	56.82 (3.73)	56.27 (4.84)	0.17
Mandible_R	D1cc^&^	<75Gy	100%	100%	1	D1cc^&^	56.88 (5.22)	56.44 (6.59)	0.37
TM Joint_L	D1cc^&^	<75Gy	100%	100%	1	D1cc^&^	38.28 (9.17)	39.07 (9.42)	0.39
TM joint_R	D1cc^&^	<75Gy	100%	100%	1	D1cc^&^	40.82 (10.73)	42.72 (10.20)	0.026
Larynx	Mean	<45 Gy	48.1%	34.6%	0.07	Mean	45.94 (5.65)	47.83 (5.97)	<0.001
Cochlea_L	Mean	<50Gy	78.5%	75.0%	0.76	Mean	42.87 (11.57)	43.70 (10.65)	0.33
Cochlea_R	Mean	<50Gy	75%	76.9%	1	Mean	44.21 (11.83)	42.55 (11.89)	0.24
Body						V15	3054 (771)	2976 (766)	<0.001
						V20	2633 (662)	2523 (650)	<0.001
						V25	2208 (571)	2095 (555)	<0.001
						V30	2178 (593)	1679 (495)	<0.001

Abbreviations: IMRT, intensity modulated radiation therapy; VMAT, volumetric modulated arc therapy; OAR, organ at risk; PRV, planning risk volume; L, light; R, right

¶ The dose received by 1% of the volume.

§ The percentage volume of at least one gland which received >30 Gy.

* The volume of both glands which received < 20 Gy.

&: Dose received by 1cubic centimeter of the volume.

Dose optimization and calculation for s-IMRT and VMAT plan were performed on the Monaco treatment planning system (version 3.02; Elekta Medical Systems, Crawley, UK) using the Monte Carlo algorithm. Both IMRT and VMAT were generated for an Elekta Synergy linear accelerator using 6 MV photons, equipped with the Elekta Precise Beam VMAT® linac control system and a conventional 80 leaf MLC (1 cm leaf width at iso-center). All plans were generated by a team of dosimetrists who experienced in planning s-IMRT and VMAT by using a whole-field (including neck-radiation) simultaneous integrated-boost technique(1). IMRT plan: A standard coplanar 9F gantry arrangement was designed in all cases and delivered in the step-and-shoot mode(2). VMAT: All plans used a single complementary coplanar arc of 360° with the couch angle set to 0°.

### Planning comparison

Quantitative evaluation of the PTVs was performed using a standard Dose-Volume Histogram (DVH), according to ICRU 83. The dose values covering 98% and 2% of the PTV (D98 and D2, respectively) were calculated as metrics for the minimum and maximum doses, in addition to the mean dose (D50). The conformity index (CI), a measure of target volume dose distribution conformity, was calculated using CI = TVRI/TV × TVRI/VRI (TVRI, target volume covered by reference isodose; TV, target volume; VRI, volume of the reference isodose). The homogeneity index (HI), a measure of the evenness of dose distribution, was calculated using HI = (D2 - D98) / D50, where D2, D98 and D50 are the doses covering 2%, 98% and 50% of the PTV, respectively.

Analysis of the OARs included the maximum dose, mean dose and a set of appropriate define (Vx) and define (Dy) values. Focus was also placed on the low dose burden of healthy tissue, calculated as the volumes of healthy tissue receiving more than 15, 20, 25, 30 Gy.

Treatment time (defined as the time from beam-on to beam-off, including radiation delivery and gantry rotation, and excluding patient setup and image comparison) and monitoring time were calculated using the Elekta Synergy.

### Statistical analysis

Statistical analysis was performed using SPSS 17.0 (SPSS Inc., Chicago, IL). The differences between VMAT and s-IMRT were compared using the T matched-pair signed-rank test and the pass-rate was compared using McNemar test. Two-tailed *P* values < 0.05 were considered significant.

## Results

### The dose distrbution of the PTVs

**Figure 1:**
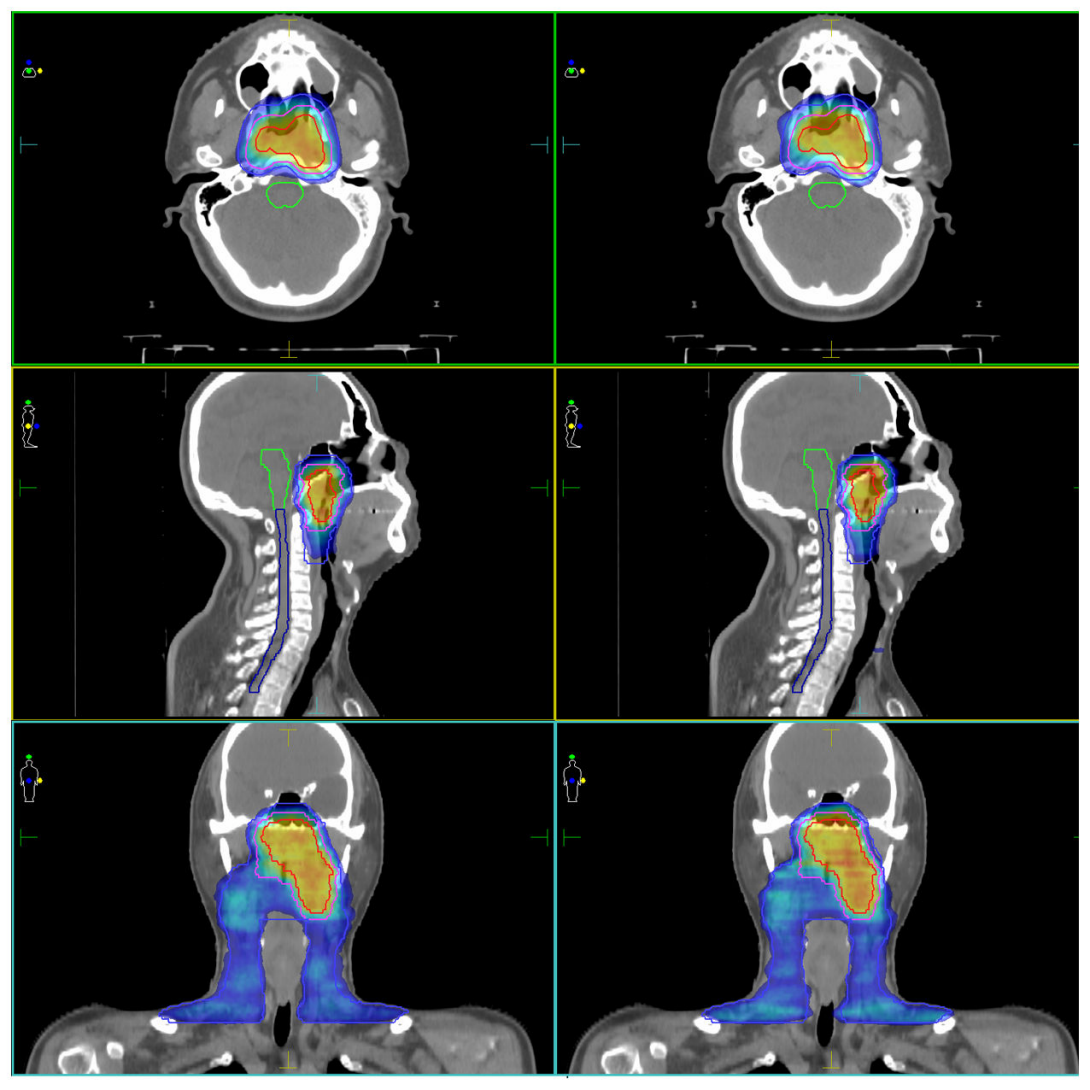
Isodose distributions for a representative NPC patient (stage cT1N2M0) in the volumetric modulated arc therapy (VMAT) plan (left) and step and shoot intensity modulated radiation therapy (s-IMRT) plan (right), indicating the PTV-7000 (red), PTV-6000 (pink), PTV-5600 (light blue).

Both strategies met the dose objectives for the OARs in most patients, except for several T4 patients where neither the VMAT nor s-IMRT plans met the OARs dose objectives. Coverage of the PTVs by VMAT and s-IMRT was similar and excellent; the average coverage of 95% of the PTV70 was 100% of the prescribed dose for all IMRT and VMAT plans. Target coverage by IMRT and VMAT was not significantly different (Table 1). S-IMRT had generally superior CI and HI for the PTVs, compared to VMAT (Table 1). Isodose distributions for a representative NPC patient (stage cT1N2M0) in the VMAT and s-IMRT plan were shown in Figure 1.

### The dose distrbution of Sparing OARs

**Figure 2:**
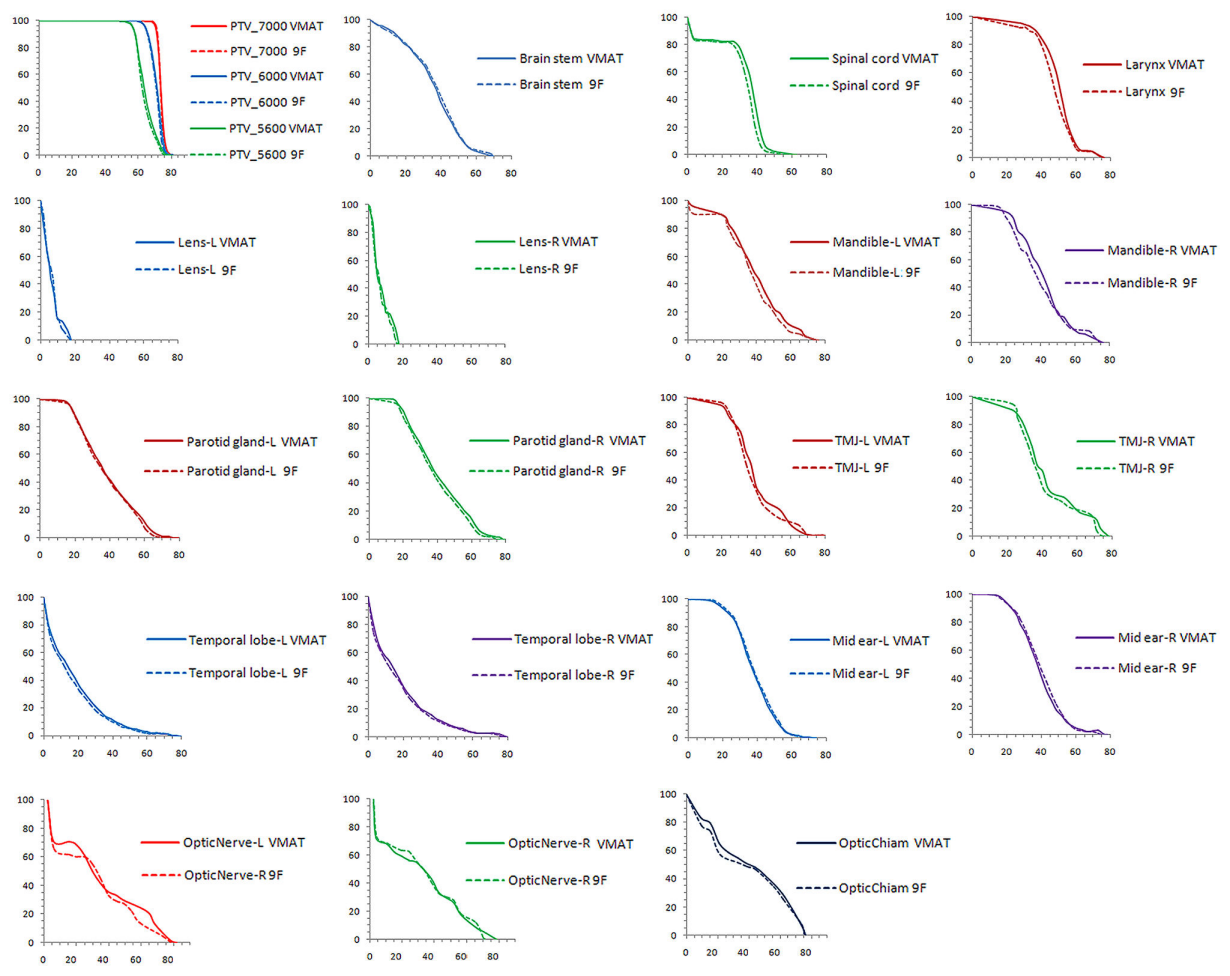
Average dose volume histograms for all 52 NPC patients according to the step and shoot intensity modulated radiation therapy (s-IMRT) plans and volumetric modulated arc therapy (VMAT) plans.

All of IMRT and VMAT plans which met the dose objectives for the OARs were similar; however, only 15 patients met the V30 < 50% planning objective for the parotid glands, regardless of the strategy. Notably, s-IMRT resulted in a slightly lower dose for the spinal cord, lens and larynx (Table 2) and higher D1 for the brainstem. The irradiation received by most other structures was similar using both techniques (Figure 2). However, the low dose burden values achieved with VMAT were significantly lower than s-IMRT (Table 2).

### Stratified analysis of the PTVs by T category

In order to compare the dosimetric characteristics within each T-classification, the patients were divided into four groups: T1, T2, T3 and T4. The D2 of PTVs for VMAT plans was higher than s-IMRT plans in all patient groups; this difference became more obvious as T classification increased (Table 3; all *P* < 0.05). VMAT lead to a lower HI in all patient groups, compared to s-IMRT (*P* < 0.05). The CI of IMRT and VMAT plans were similar in T1-2 patients; however the CI of VMAT plans was lower in T3-4 patients than IMRT plans (Table 3; *P* < 0.05).

**Table 3:** Mean difference in dose between s-IMRT and VMAT of s-IMRT and VMAT for the PTVs in 52 NPC patients stratified by T category.

		**T1**		**T2**		T3		T4	
**Target**	**Index**	**Difference** ^‡^ **(Gy**)	***P***	**Difference** ^‡^ **(Gy**)	***P***	**Difference** ^‡^ **(Gy**)	***P***	**Difference** ^‡^ **(Gy**)	***P***
**PTV_7000**	**D2** ^¶^	0.52±0.74	0.07	0.59±1.27	0.197	1.79±1.44	<0.001	2.21±1.40	<0.001
	**D50** ^§^	-0.03±0.46	0.84	0.21±1.26	0.63	0.30±0.63	0.03	0.54±0.82	0.05
	**D98** ^&^	-0.52±0.64	0.04	-0.91±0.95	0.02	-0.39±0.54	0.002	0.03±0.76	0.89
	**CI**	-0.03±0.04	0.107	-0.01±0.08	0.85	-0.09±0.09	<0.001	-0.10±0.06	<0.001
	**HI**	0.01±0.01	<0.001	0.02±0.15	0.004	0.03±0.02	<0.001	0.03±0.02	0.001
**PTV_6000**	**D2** ^¶^	0.53. ±0.67	0.05	0.53±1.25	0.24	1.23±1.15	<0.001	2.35±1.29	<0.001
	**D50** ^§^	-0.15±0.58	0.46	-1.1±3.09	0.32	0.34±0.59	0.012	0.06±0.46	0.001
	**D98** ^&^	-0.55±1.29	0.68	-0.62±1.29	0.19	-0.36±0.76	0.036	0.82±0.61	0.001
	**HI**	0.01±0.02	0.20	0.02±0.03	0.05	0.02±0.02	<0.001	0.02±0.02	0.004
**PTV_5600**	**D2** ^¶^	0.37±0.73	0.16	0.64±1.25	0.16	0.80±0.80	<0.001	1.43±1.70	0.02
	**D50** ^§^	0.55±1.11	0.17	0.67±1.30	0.16	0.11±0.98	<0.001	1.90±1.54	0.002
	**D98** ^&^	-1.29±0.32	0.004	-1.00±0.97	0.02	-0.83±0.91	<0.001	-0.66±0.76	0.02
	**HI**	0.02±0.01	0.01	0.02±0.02	0.02	0.02±0.02	<0.001	0.02±0.03	0.03

Abbreviations: PTV, planning target volume; CI, conformity index; HI, homogeneity index; IMRT, intensity modulated radiation therapy; VMAT, volumetric modulated arc therapy.

¶ Dose received by 2% of the volume.

§ Dose received by 50% of the volume.

&: Dose received by 98% of the volume.

‡ refer to VMAT minus s-IMRT values

### Stratified analysis of the OARs by T category

In patients with early T1-2 disease, the dose delivered by both techniques was similar and VMAT resulted in better sparing of the brainstem (Table 4; *P* < 0.05). However, in patients with an advanced T-classification, VMAT plans resulted in significantly poorer sparing of the spinal cord, bilateral optic nerve, optic chiasm, lens, parotid gland, TM joint, and larynx than s-IMRT plans (Table 4; all *P* < 0.05).

**Table 4:** Mean differences in dose between s-IMRT and VMAT for the organs at risk (OARs) in 52 NPC patients stratified by T category.

		**T1**		**T2**		**T3**		**T4**	
**OAR**	**Index**	**Difference** ^‡^ **(Gy**)	***P***	**Difference** ^‡^ **(Gy**)	***P***	**Difference** ^‡^ **(Gy**)	***P***	**Difference** ^‡^ **(Gy**)	***P***
**Brainstem_PRV**	D1^¶^	-1.81±2.33	0.04	-1.01±2.09	0.19	-0.05±1.92	0.89	1.20±1.94	0.07
**SpinalCord_PRV**	D1^¶^	0.66±1.82	0.31	1.47±2.75	0.15	2.21±1.60	<0.001	2.69±2.12	0.002
**OpticNerves_L**	D1^¶^	0.26±6.57	0.91	-2.42±7.70	0.15	4.49. ±9.92	0.041	3.60. ±4.19	0.02
**OpticNerves_R**	D1^¶^	0.73±2.43	0.40	-0.15±6.18	0.94	2.55±4.94	0.022	3.04±3.40	0.01
**Chiasm**	D1^¶^	2.72±3.65	0.06	0.58±7.23	0.82	2.92±4.36	0.005	2.87±3.76	0.03
**Lens_L**	max	1.07±1.65	0.86	0.17±0.94	0.59	0.66±1.29	0.022	0.71±0.99	0.04
**Lens_R**	max	0.76±1.32	0.12	0.64±1.22	0.15	0.70±1.43	0.028	0.10±1.99	0.87
**Parotid _L**	mean	-0.81±2.77	0.41	0.33±3.26	0.76	0.83±2.78	0.17	0.84±1.67	0.12
	D50^*^	0.02±4.48	0.99	-0.78±4.90	0.65	0.22±3.60	0.78	-0.13±2.39	0.86
	V30^§^	0.22±8.41	0.94	1.84±10.79	0.65.	1.96±8.94	0.31	1.30±7.61	0.59
**Parotid _R**	mean	-0.07±2.50	0.94	0.75±5.28	0.42	1.34±2.60	0.022	1.15±2.36	0.14
	D50^*^	0.10±3.40	0.93	-0.76±7.08	0.75	1.44±2.87	0.025	0.89±2.73	0.30
	V30^§^	0.70±8.67	0.82	1.55±11.93	0.39	3.17±8.90	0.10	-2.22±6.65	0.29
**TemporalLobe_L**	D1^¶^	0.96±5.44	0.61	-0.86±3.07	0.42	-1.06±6.26	0.42	1.39±2.66	0.11
**TemporalLobe_R**	D1^¶^	-1.13±5.01	0.52	-0.47±2.68	0.62	1.36±3.43	0.070	2.20±2.75	0.02
**Mandible_L**	D1cc^&^	-3.46±3.06	0.009	-0.04±3.56	0.97	-0.17±2.08	0.70	0.63±2.01	0.32
**Mandible_R**	D1cc^&^	-2.59±2.94	0.03	-0.24±4.54	0.88	-0.57±2.55	0.30	1.74±3.84	0.16
**TM Joint_L**	D1cc^&^	-0.04±4.85	0.98	-2.72±10.54	0.46	1.99±6.23	0.14	1.84±3.64	0.13
**TM Joint_R**	D1cc^&^	2.61±3.99	0.09	-2.14±9.97	0.54	3.21±14.35	0.002	1.43±5.21	0.38
**Larynx**	mean	0.26±2.29	0.74	1.74±23.48	0.17	3.45±3.22	<0.001	-0.17±3.65	0.88
**Cochlea_L**	mean	1.78±5.49	0.36	-1.50±4.09	0.30	1.85±7.53	0.25	0.47±4.80	0.75
**Cochlea_R**	mean	-1.03±4.65	0.53	-2.75±4.72	0.12	0.47±7.42	0.76	0.12±2.48	0.88
**Body**	V15(cc)	-68.82±141.32	0.18	-34.57±140.78	0.48	-95.60±117.07	0.001	-81.90±54.42	0.001
	V20(cc)	-112.54±117.74	0.02	-39.01±147.17	0.45	-138.43±126.44	<0.001	-107.91±41.62	<0.001
	V25(cc)	-81.14±155.16	0.13	-51.90±102.55	0.17	-158.60±130.87	<0.001	-90.00±82.39	0.005
	V30(cc)	-463.92±214.87	<0.001	-494.98±234.73	<0.001	-545.30±186.70	<0.001	-437.73±95.25	<0.001

¶ The dose received by 1% of the volume.

* The dose received by 50% of the volume.

§ Volume of at least one gland which received > 30 Gy.

&: Dose received by 1 cc of the volume.

‡: refer to VMAT minus s-IMRT values.

### Monitor units and delivery time

The monitor units (MUs) for the VMAT and s-IMRT plans were similar (VMAT vs. s-IMRT, 644 ± 172 vs. 654 ± 180; *P* > 0.05), with VMAT leading to an average MU reduction of 2% (*P* > 0.05). The average treatment time for VMAT, as measured manually during treatment delivery, was 424s ± 64 s compared to 778 ± 126 s for s-IMRT (*P* < 0.05).

## Discussion

Previous planning studies have reported that VMAT techniques achieved similar or higher quality treatment plans with fewer MU and a shorter delivery time, compared to conventional IMRT [11,12,15]. To the best of our knowledge, this dosimetric comparison of VMAT plans, generated using the Monaco system with s-IMRT plans is the largest cohort for NPC patients, and performed stratified analysis for each group.

### Dose comparison of IMRT and VMAT plans

VMAT plans achieved similar target volume coverage as IMRT plans. All VMAT plans met the target coverage planning goals (Table 1). Johnston et al. reported D98 values of 98.0% and 95.8% of the prescribed dose for PTV70 and PTV56 in five NPC patients, and Vanetti et al. reported an average D98 of 93.2% of the prescribed target area dose in 29 cases of oropharyngeal, hypopharyngeal or laryngeal carcinoma [15,16]. This study achieved average PTV70 and PTV56 values of 99.2 and 97.7% and a higher D98 of the prescribed dose, indicating superior target coverage compared to previous studies. The PTV70 dose tended to have a lower conformity and was less homogeneous using VMAT compared to IMRT, in agreement with Johnston et al. who reported that the target conformity of VMAT (1.91) was slightly poorer than IMRT (2.32) [16].

However, low dose volumes (volume receiving > 15 Gy, 20Gy, 25Gy, 30Gy) of healthy tissues were significantly lower with VMAT than s-IMRT plans; partly due to the fact that VMAT delivers the total dose over a larger number of angles. The reduced low dose volumes offered by VMAT may reduce the risk of the second cancer in healthy tissue, and this is worthy of further investigation in future studies.

Large reductions in the dose to the OARs have been reported when shifting from IMRT to rapid arc VMAT; however, a large dose reduction was not evident in the OARs in this study. This is the first dosimetric comparison of s-IMRT and VMAT using the Monaco TPS based on biological optimization in a large number of NPC patients. Unlike other head and neck malignancies, NPC requires more sufficient sparing of critical normal structures, necessitating a larger and more superior treatment strategy, and also encompasses the entire neck lymph nodal chain down to the supraclavicular fossa. Thus, treatment planning for NPC is challenging.

It is difficult to compare planning studies at different institutions due to variations in prescribing, optimization methods and systems, dosimetric parameter reporting and planning systems. Additionally, the dosimetric results depend on the location and approach to the target(s), OARs contouring, extent of CTV expansion and dosimetric endpoint, in agreement with the results of Guckenberger et al. [17]. The differences between VMAT and s-IMRT observed are not only related to the VMAT plans, but also to the standard s-IMRT treatment used as a baseline for comparison. In terms of the dose to the OARs, the VMAT and IMRT plans had a similar success for achieving the dose constraints (Table 1), although there were significant differences in the values obtained using each technique. The parotid gland and larynx doses did not meet the dose constraints, due to their partial overlap with the PTV and the requirement for upper cervical nodal coverage in the higher intermediate-dose volumes. Furthermore, similarly to the study of Johnston et al., the contouring of the parotid gland in this study included the deep leaves [16].

Stratification analysis revealed that the dose distribution of the VMAT plans was similar to s-IMRT in T1-2 patients. In T3-4 patients, VMAT lead to poorer sparing in the normal tissue dose distribution. However, in patients with an advanced T-classification, the doses received by the optic nerve, optic chiasm and lens dose in the VMAT plans were significantly higher than the IMRT plans. Based on clinical experience, patients are placed in a over-supine position on the premise of comfort. With this position, the dose received by the optic nerves and chiasm significantly decreased, which may be related to the fact that the tumor is positioned close to the optic nerve and optic chiasm in T3-4 patients with bony erosion.

### VMAT reduces MU and delivery time

Only a modest reduction was observed in the MU per fraction using VMAT. Previous investigators have reported large reductions of more than 600 MU per fraction using rapid arc [11,15]. However, the MU per fraction for the single arc VMAT plans used in this study are similar to the MU of 586, 584 and 672.4 MU reported by previous reports for single and double arc plans in NPC and other head and neck cancers [15,16,18]. Thus, the varied MU reduction observed in different studies is mainly related to the number of MU in the IMRT plans, and not to the number of MU in the VMAT plans, possibly due to differences in the IMRT optimization algorithms and techniques used (e.g. sliding window vs. step-and-shoot).

The delivery efficiency of VMAT in this study (424 s) was slightly longer than previous reports (90-265 s); however, these studies used rapid arc (Varian) and smart arc (Pinnacle) VMAT. Wiezorek et al. [19] demonstrated that the delivery time for double arc VMAT delivered via Elekta (9 min) was longer than rapid arc VMAT (2 min) in a multi-center system. The delivery efficiency of VMAT is mainly related to the binary dose rate of Elekta accelerators. Shorter treatment times will increase patient compliance and the accuracy of treatment due to reduced intra-fractional patient motion. Moreover, the time gained can be used to increase patient throughput, especially at our hospital, or to increase image guidance which could facilitate margin reduction and thereby reduce toxicity [20].

## Conclusions

VMAT provides similar dose coverage of the PTVs and similar or better normal tissue sparing than s-IMRT in early T category NPC; however, VMAT leads to poorer OARs sparing in advanced T category NPC. The delivery time required for VMAT is significantly shorter than s-IMRT.
